# Incident Clinical and Mortality Associations of Myocardial Native T1 in the UK Biobank

**DOI:** 10.1016/j.jcmg.2022.06.011

**Published:** 2023-04

**Authors:** Zahra Raisi-Estabragh, Celeste McCracken, Evan Hann, Dorina-Gabriela Condurache, Nicholas C. Harvey, Patricia B. Munroe, Vanessa M. Ferreira, Stefan Neubauer, Stefan K. Piechnik, Steffen E. Petersen

**Affiliations:** a William Harvey Research Institute, NIHR Barts Biomedical Research Centre, Queen Mary University London, Charterhouse Square, London, United Kingdom; b Barts Heart Centre, St Bartholomew’s Hospital, Barts Health NHS Trust, West Smithfield, London, United Kingdom; c Division of Cardiovascular Medicine, Radcliffe Department of Medicine, University of Oxford, National Institute for Health Research Oxford Biomedical Research Centre, Oxford University Hospitals NHS Foundation Trust, Oxford, United Kingdom; d Oxford Centre for Clinical Magnetic Resonance Research (OCMR), Division of Cardiovascular Medicine, British Heart Foundation Centre of Research Excellence, Oxford NIHR Biomedical Research Centre, University of Oxford, United Kingdom; e London North West University Healthcare NHS Trust, Watford Road, Harrow, United Kingdom; f MRC Lifecourse Epidemiology Centre, University of Southampton, Southampton, United Kingdom; g NIHR Southampton Biomedical Research Centre, University of Southampton and University Hospital Southampton NHS Foundation Trust, Southampton, United Kingdom; h National Institute for Health Research Oxford Biomedical Research Centre, Oxford University Hospitals NHS Foundation Trust, Oxford, United Kingdom; i Health Data Research UK, London, United Kingdom; j Alan Turing Institute, London, United Kingdom

**Keywords:** cardiovascular disease, cardiac magnetic resonance, incident events, mortality, native T1 mapping

## Abstract

**Background:**

Cardiac magnetic resonance native T1-mapping provides noninvasive, quantitative, and contrast-free myocardial characterization. However, its predictive value in population cohorts has not been studied.

**Objectives:**

The associations of native T1 with incident events were evaluated in 42,308 UK Biobank participants over 3.17 ± 1.53 years of prospective follow-up.

**Methods:**

Native T1-mapping was performed in 1 midventricular short-axis slice using the Shortened Modified Look-Locker Inversion recovery technique (WIP780B) in 1.5-T scanners (Siemens Healthcare). Global myocardial T1 was calculated using an automated tool. Associations of T1 with: 1) prevalent risk factors (eg, diabetes, hypertension, and high cholesterol); 2) prevalent and incident diseases (eg, any cardiovascular disease [CVD], any brain disease, valvular heart disease, heart failure, nonischemic cardiomyopathies, cardiac arrhythmias, atrial fibrillation [AF], myocardial infarction, ischemic heart disease [IHD], and stroke); and 3) mortality (eg, all-cause, CVD, and IHD) were examined. Results are reported as odds ratios (ORs) or HRs per SD increment of T1 value with 95% CIs and corrected *P* values, from logistic and Cox proportional hazards regression models.

**Results:**

Higher myocardial T1 was associated with greater odds of a range of prevalent conditions (eg, any CVD, brain disease, heart failure, nonischemic cardiomyopathies, AF, stroke, and diabetes). The strongest relationships were with heart failure (OR: 1.41 [95% CI: 1.26-1.57]; *P =* 1.60 × 10^-9^) and nonischemic cardiomyopathies (OR: 1.40 [95% CI: 1.16-1.66]; *P =* 2.42 × 10^-4^). Native T1 was positively associated with incident AF (HR: 1.25 [95% CI: 1.10-1.43]; *P =* 9.19 × 10^-4^), incident heart failure (HR: 1.47 [95% CI: 1.31-1.65]; *P =* 4.79 × 10^-11^), all-cause mortality (HR: 1.24 [95% CI: 1.12-1.36]; *P =* 1.51 × 10^-5^), CVD mortality (HR: 1.40 [95% CI: 1.14-1.73]; *P =* 0.0014), and IHD mortality (HR: 1.36 [95% CI: 1.03-1.80]; *P =* 0.0310).

**Conclusions:**

This large population study demonstrates the utility of myocardial native T1-mapping for disease discrimination and outcome prediction.

Cardic magnetic resonance (CMR) is the reference standard for evaluation of cardiac structure and function. CMR myocardial native T1 mapping provides quantitative, noninvasive, and contrast-free characterization of myocardial tissue on a pixel-by-pixel basis, comparable to a virtual biopsy of the living heart.^1^^,^^2^

The clinical utility of myocardial native T1 mapping has been shown in select clinical cohorts, particularly for the diagnosis of acute myocardial injury, myocardial inflammation, myocardial iron overload, Fabry disease, and cardiac amyloidosis.^3^ However, the prognostic value of native T1 in large population cohorts without pre-existing disease has not been previously studied.

The UK Biobank is a very large population-based cohort study including detailed CMR and prospective tracking of incident health events through linkages to routine health data.^4^

We studied demographic and clinical associations of myocardial native T1 in 42,308 UK Biobank participants. Importantly, we evaluated relationships with key incident diseases and mortality outcomes.

## Methods

### Setting and study population

The UK Biobank is a population-based cohort of more than 500,000 participants recruited between 2006 and 2010. Postal invitations were sent to individuals aged 40 to 69 years old, identified through NHS (National Health Service) registers, living within 25 miles of 1 of 22 UK Biobank assessment centers. Individuals who were unable to consent or complete baseline assessment due to ill health or discomfort were not recruited. At baseline recruitment (2006-2010), there was detailed characterization of participants’ demographic and clinical status, as well as a series of physical measures and blood sampling. The UK Biobank protocol is publicly available.^5^ The UK Biobank imaging study, which is ongoing, was launched in 2015 and aims to scan a random 20% subset of the original cohort.^4^ The imaging protocol includes detailed CMR. Linkages to national health data, such as HES (Hospital Episode Statistics) and Office for National Statistics death registration data, permit prospective tracking of incident health events for all UK Biobank participants.

### Ethics statement

This study complies with the Declaration of Helsinki; the work was covered by the ethical approval for UK Biobank studies from the NHS National Research Ethics Service on June 17, 2011 (Ref 11/NW/0382) and extended on June 18, 2021 (Ref 21/NW/0157) with written informed consent obtained from all participants.

### CMR image acquisition and analysis

CMR scans were performed using 1.5-T scanners (MAGNETOM Aera, Syngo Platform VD13A, Siemens Healthcare) in dedicated imaging centers with uniform equipment and staff training. The predefined UK Biobank acquisition protocol is available in a separate publication.^6^ Myocardial native T1 mapping was acquired in 1 midventricular short-axis slice using the ShMOLLI (Shortened Modified Look-Locker Inversion) recovery technique (WIP780B). The typical pulse sequence parameters are as published previously by Piechnik et al.^7^ Global myocardial native T1 was calculated from the entire short-axis slice using a fully automated quality-controlled analysis tool, excluding studies with a predicted Dice score of <0.7; technical details of the tool, including comparison of the manual and automated T1 measures, are described elsewhere.^8^

### Ascertainment of clinical and mortality outcomes

Diseases were defined based on a combination of UK Biobank baseline assessment records and HES International Classification of Disease codes (Supplemental Table 1), as per previous publications using this cohort.^9^ We included the following prevalent outcomes: any cardiovascular disease (CVD), any brain disease, valvular heart disease, heart failure, nonischemic cardiomyopathies, cardiac arrhythmias, atrial fibrillation (AF), myocardial infarction (MI), ischemic heart disease (IHD), stroke, hypertension, diabetes, and high cholesterol. Mortality outcomes were defined according to the primary cause of death ascertained from death register data. We considered the following incident events: AF, heart failure, stroke, MI, IHD, all-cause mortality, CVD mortality, and IHD mortality.

### Statistical analysis

Statistical analysis was performed using R version 4.0.3 and RStudio Version 1.3.1093. We first examined myocardial native T1 values in a subset of healthy individuals (n = 19,297), stratified by age and sex. Healthy status was defined as the absence of any CVD or classic vascular risk factors (eg, diabetes, hypertension, high cholesterol, and smoking) at the time of imaging. We took age as recorded at the imaging visit and sex from self-report. Within the healthy subset, we estimated the association of T1 with age using linear regression models separately for men and women. We observed a sex differential trend of T1 with aging within the healthy subset. Thus, subsequent models are adjusted for age, sex, and age × sex.

We estimated associations of myocardial native T1 in the entire cohort with prevalent disease and incident events (eg, incident CVDs and mortality outcomes) using logistic regression and Cox proportional hazards regression, respectively. We investigated sex and age differential relationships of the associations with incident diseases and mortality using interaction terms added to models (T1 × age; T1 × sex) and stratified analyses by sex and median age where indicated by a significant interaction term. We examined for potential nonlinearity by examining associations in strata of above/below the median T1 value. Prevalent diseases were considered as those present at time of imaging. Incident events were considered as first occurrence of the disease after imaging; that is, individuals with record of an outcome of interest before the index date were excluded from the analysis of that outcome. We selected hematocrit, body mass index (BMI), and heart rate as potential confounders of myocardial native T1, as per previous work.^7^ BMI and average heart rate were taken from the imaging visit; hematocrit percentage was measured at baseline recruitment. In secondary analyses, we included additional adjustment for BMI, hematocrit, and heart rate. Effect estimates are expressed as odds ratios (ORs) and HRs per 1 SD increment of T1, and standardized beta coefficients and 95% CIs. We present *P* values corrected for multiple testing using the Benjamini-Hochberg procedure setting the false discovery rate to 5%.^10^ The study is reported in accordance with the STROBE (Strengthening the Reporting of Observational Studies in Epidemiology) statement (Supplemental Material).

## Results

### Participant characteristics

Myocardial native T1 was available for 42,894 participants. From these, 586 studies (0.01%) with Dice score <0.7 were excluded. Thus, 42,308 participants with analyzable native T1 were included in the analysis (Supplemental Figure 1); average age was 64.0 ± 7.7 years and 51.9% (n = 21,963) were women. For creation of the healthy subset (n = 19,297), individuals with any CVD (n = 4,855) or vascular risk factors (n = 18,126) were further excluded.

Within the whole sample (n = 42,308), the rates of diabetes, hypertension, and high cholesterol were 6.0% (n = 2,528), 33.4% (n = 14,136), and 35.6% (n = 15,041), respectively (Table 1). There was record of CVD for 11.5% (n = 4,885) of participants. As expected, IHD was the most common CVD (6.2%, n = 2,604).

**Table 1:** Participant Characteristics, Prevalent Disease, and Incident Events

	Total (N = 42,308)	Men (n = 20,345, 48%)	Women (n = 21,963, 52%)
Age at imaging, y	64.0 ± 7.7	64.8 ± 7.8	63.4 ± 7.6
BMI, kg/m^2^	25.9 (23.5-28.8)	26.4 (24.3-29.0)	25.2 (22.7-28.5)
Heart rate, beats/min	62.6 ± 10.5	61.5 ± 10.7	63.7 ± 10.2
Hematocrit, %	41.1 ± 3.5	43.3 ± 2.8	39.0 ± 2.7
Myocardial native T1, ms	932.3 ± 35.7	919.6 ± 33.0	943.9 ± 34.0
Prevalent disease			
Hypertension	14,136 (33.4)	8,231 (40.5)	5,905 (26.9)
Diabetes	2,528 (6.0)	1,606 (7.9)	922 (4.2)
High cholesterol	15,041 (35.6)	8,683 (42.7)	6,358 (28.9)
Any cardiovascular disease	4,885 (11.5)	3,070 (15.1)	1,815 (8.3)
Any brain disease	2,967 (7.0)	1,541 (7.6)	1,426 (6.5)
Valvular heart disease	802 (1.9)	409 (2.0)	393 (1.8)
Heart failure	278 (0.7)	220 (1.1)	58 (0.3)
Nonischemic cardiomyopathies	104 (0.2)	74 (0.4)	30 (0.1)
Cardiac arrhythmias	2,271 (5.4)	1,397 (6.9)	874 (4.0)
Atrial fibrillation	753 (1.8)	534 (2.6)	219 (1.0)
Myocardial infarction	1,061 (2.5)	854 (4.2)	207 (0.9)
IHD	2,604 (6.2)	1,851 (9.1)	753 (3.4)
Stroke	852 (2.0)	568 (2.8)	284 (1.3)
Incident events			
CVD (any)	1,256 (3.0)	788 (3.9)	468 (2.1)
AF	215 (0.5)	145 (0.7)	70 (0.3)
Stroke	215 (0.5)	132 (0.6)	83 (0.4)
MI	241 (0.6)	169 (0.8)	72 (0.3)
IHD	649 (1.5)	430 (2.1)	219 (1.0)
Heart failure	243 (0.6)	162 (0.8)	81 (0.4)
All-cause mortality	402 (1.0)	263 (1.3)	139 (0.6)
CVD mortality	76 (0.2)	56 (0.3)	20 (0.1)
IHD mortality	44 (0.1)	36 (0.2)	8 (0.0)

Values are mean ± SD, median (IQR), or n (%).

AF = atrial fibrillation; BMI = body mass index; CVD = cardiovascular disease; IHD = ischemic heart disease; MI = myocardial infarction.

Over a follow-up period of 3.17 ± 1.53 years, we observed 402 (1.0%) deaths; of these, 76 were attributed to CVD and 44 to IHD (Table 1). The most common incident diseases were IHD (n = 649, 1.5%) and heart failure (n = 243, 0.6%). There were 241 (1.0%) incident MIs and 215 (0.5%) incident cases each of AF and stroke (Table 1).

### Myocardial native T1 in healthy participants

**Figure 1:**
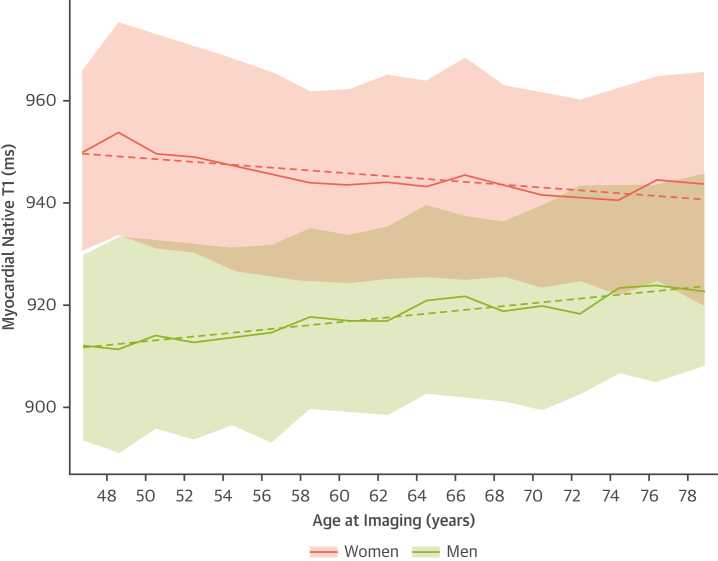
Median Myocardial Native T1 by Age and Sex in the Healthy Subset **Shaded areas** indicate the middle 50% (25th percentile to 75th percentile). The **solid line** is the median myocardial native T1 within 2-year age groups stratified by sex. The **dashed line** shows the linear trend of T1 by age, stratified by sex. The **median points** at the left-most group reflect the median T1 among participants 44 to 48 years of age. The **final median points** at right-most group reflect the median T1 among participants 78 to 84 years of age.

Within the healthy subset, women had, on average, higher myocardial native T1 than men across all age groups (range: 44 to 84 years), with the greatest difference at younger ages (Figure 1, Table 2). With increasing age, myocardial native T1 decreased in women (beta = −0.33 [95% CI: −0.41 to −0.24]; *P <* 0.0001) and increased in men (beta = 0.48 [95% CI: 0.39-0.57]; *P <* 0.0001) (Figure 1, Supplemental Table 2).

**Table 2:** Global Myocardial Native T1 in Healthy Men and Women Stratified by Age

Age Group, y	Global Myocardial T1, ms	
	Women (n = 11,479)	Men (n = 7,818)
44-45	952.1 ± 35.0	913.2 ± 31.2
55-64	945.3 ± 33.1	917.2 ± 31.1
65-74	944.8 ± 33.5	921.2 ± 32.6
75-84	944.5 ± 33.1	926.4 ± 31.9
Overall mean	946.6 ± 33.7	918.3 ± 31.9

Values are mean ± SD.

### Associations of myocardial native T1 with prevalent disease

Within the entire cohort, higher myocardial native T1 was associated with significantly greater odds of any CVD, any brain disease, heart failure, nonischemic cardiomyopathies, cardiac arrhythmias, AF, stroke, and diabetes (Central Illustration, Table 3). Of these, the largest effect sizes were observed with heart failure (OR: 1.41 [95% CI: 1.26-1.57]; *P <* 0.0001) and nonischemic cardiomyopathies (OR: 1.40 [95% CI: 1.16-1.66]; *P =* 0.0002). Hypertension and high cholesterol were associated with significantly lower myocardial native T1, with a larger effect size observed with hypertension (OR: 0.88 [95% CI: 0.86-0.90]; *P <* 0.0001).

**Central Illustration:**
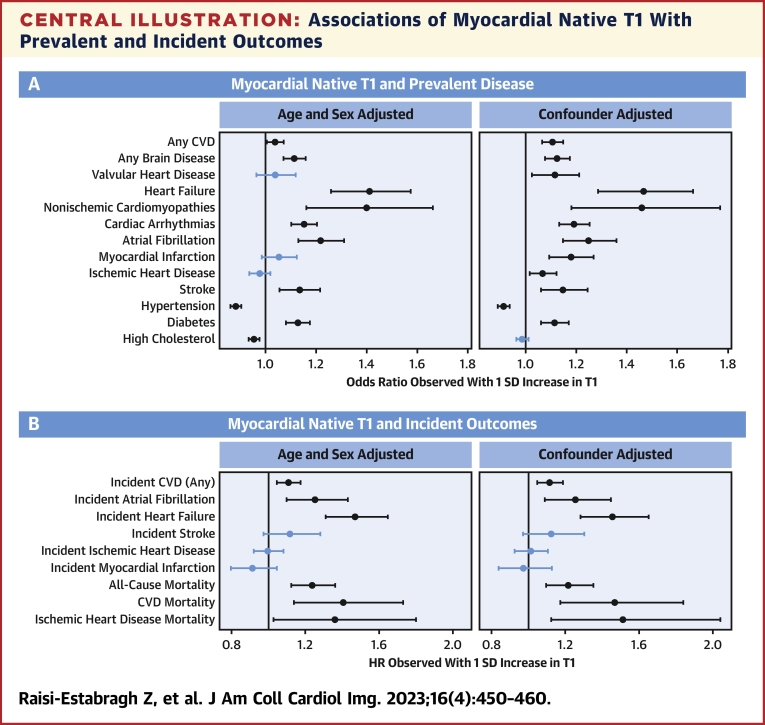
Associations of Myocardial Native T1 With Prevalent and Incident Outcomes **(A)** Results are odds ratios from logistic regression models. **(B)** Results are HRs from Cox hazards proportional regression models. The diseases listed are set as the model outcome (response variable) and native T1 is the exposure of interest. The “age and sex adjusted models” are adjusted for age, sex, and age × sex. The “confounder adjusted” models are adjusted for age, sex, age × sex, hematocrit, body mass index, and heart rate. Each bar corresponds to a separate model. The point estimate and 95% CI are indicated by the **point** and **bars**, respectively. The **blue bars** indicate statistically nonsignificant associations. CVD = cardiovascular disease.

**Table 3:** Associations of Myocardial Native T1 With Prevalent Disease

	OR (95% CI)	*P* Value
Any CVD	1.04^a^ (1.00-1.07)	0.0260
Any brain disease	1.11^a^ (1.07-1.16)	<0.0001
Valvular heart disease	1.04 (0.96-1.12)	0.3094
Heart failure	1.41^a^ (1.26-1.57)	<0.0001
Nonischemic cardiomyopathies	1.40^a^ (1.16-1.66)	0.0002
Cardiac arrhythmias	1.15^a^ (1.10-1.20)	<0.0001
AF	1.22^a^ (1.13-1.31)	<0.0001
MI	1.05 (0.99-1.12)	0.1243
IHD	0.98 (0.93-1.02)	0.2839
Stroke	1.13^a^ (1.06-1.22)	0.0005
Hypertension	0.88^a^ (0.86-0.90)	<0.0001
Diabetes	1.13^a^ (1.08-1.18)	<0.0001
High cholesterol	0.95^a^ (0.93-0.98)	0.0001

Results are from logistic regression models with diseases of interest set as the model outcome (response variable). Native T1 is the exposure of interest. There is adjustment for age, sex, and age × sex. The effect estimates as expressed as OR per 1 SD increase in T1 (ie, change in odds of outcome per 1 SD = 35.7-ms increment in native T1) with corresponding 95% CI and *P* values.

OD = odds ratio; other abbreviations as in Table 1.

a Statistically significant result following multiple testing adjustment with a false discovery rate of 0.05.

### Associations of myocardial native T1 with incident disease and mortality

Within the entire cohort, higher myocardial native T1 was associated with significantly greater hazard of incident heart failure (HR: 1.47 [95% CI: 1.31-1.65]; *P <* 0.0001) and incident AF (HR: 1.25 [95% CI: 1.10-1.43]; *P =* 0.0009). Higher myocardial native T1 was also associated with significantly greater hazard of all-cause mortality, CVD mortality, and IHD mortality (Central Illustration, Table 4). There were no statistically significant associations of native T1 with incident MI, incident IHD, or incident stroke (Central Illustration, Table 4).

**Table 4:** Associations of Myocardial Native T1 With Incident Events

	HR (95% CI)	*P* Value
Incident CVD (any)	1.11^a^ (1.05-1.17)	0.0005
Incident AF	1.25^a^ (1.10-1.43)	0.0009
Incident stroke	1.12 (0.97-1.28)	0.1191
Incident IHD	1.00 (0.92-1.08)	0.9368
Incident MI	0.91 (0.80-1.05)	0.1871
Incident heart failure	1.47^a^ (1.31-1.65)	<0.0001
All-cause mortality	1.24^a^ (1.12-1.36)	<0.0001
CVD mortality	1.40^a^ (1.14-1.73)	0.0014
IHD mortality	1.36^a^ (1.03-1.80)	0.0310

Results are from Cox proportional hazards regression models with outcomes of interest set as the model outcome (response variable). Native T1 is the exposure of interest. There is adjustment for age, sex, and age × sex. The effect estimates as expressed as HR per 1 SD increase in T1 (ie, change in hazard of outcome 1 SD = 35.7 ms increase in native T1) with corresponding 95% CI and *P* values.

Abbreviations as in Table 1.

a Statistically significant result following multiple testing adjustment with a false discovery rate of 0.05.

### Sex/age differential patterns and potential nonlinearity

Given that associations with incident disease and mortality outcomes are of the greatest clinical interest, we additionally evaluated age and sex differential dependencies of these relationships (Supplemental Table 3). There was no evidence of a sex differential relationship for any of the incident outcomes. For the mortality outcomes, we observed evidence of a significant interaction of T1 with age. Thus, for these outcomes, we proceeded to examine associations with T1 stratified by median age (65 years); in doing so, we observed greater magnitude of association in older individuals (Supplemental Table 4). We additionally assessed for potential nonlinearity of the associations of T1 with incident outcomes, by modelling in strata above and below the median T1 value. Overall patterns suggested that higher T1 values had stronger (ie, larger magnitude of effect) associations with incident outcomes (Supplemental Table 5).

### The importance of potential confounders

Higher BMI and hematocrit were significantly associated with lower myocardial native T1, whereas faster average heart rate was associated with significantly higher native T1 (Supplemental Table 6). In models with additional confounder adjustment, all previously observed associations between higher native T1 and prevalent diseases remained robust, with additional significant relationships observed with valvular heart disease, MI, and IHD (Central Illustration, Supplemental Table 7). The negative association with hypertension remained unchanged; however, the relationship with high cholesterol was attenuated. The positive association of native T1 with IHD mortality appeared stronger; relationships with other incident outcomes were unchanged (Central Illustration, Supplemental Table 7).

## Discussion

### Summary of findings

In this large population-based cohort of 42,308 individuals, we describe associations of intrinsic myocardial tissue properties, as quantified by native T1-mapping, with key demographics, diseases, and incident health outcomes. Among healthy participants, women had, on average, higher global myocardial native T1 than men across all ages. There was a sex differential trend of myocardial native T1 with aging, with a significant positive association in men and a negative trend in women. In the whole sample, higher myocardial native T1 was associated with significantly greater likelihood of prevalent CVD, brain disease, heart failure, nonischemic cardiomyopathies, AF, stroke, and diabetes. We also observed significant positive associations between myocardial native T1 and risk of incident AF and incident heart failure. Importantly, we show significant associations of native myocardial T1 measures with all-cause mortality, CVD mortality, and IHD mortality. These relationships appeared more convincing with adjustment for potential confounders.

### Age and sex associations in healthy participants

Within the healthy subset of 11,479 women and 7,818 men, we observed higher native T1 in women than men across all age groups. This observation is consistent with multiple previous reports in healthy cohorts.^11^, ^12^, ^13^, ^14^, ^15^ These findings may reflect genuine differences in the myocardial tissue character of men and women. Technical factors likely also play a role in augmenting any differences. In particular, the lower average wall thickness in women is expected to systematically shift the measurement error in global myocardial native T1 toward higher values through partial volume effects on magnetic resonance imaging. Indeed, previous work has described the negative associations of native T1 and myocardial thickness.^7^

There are inconsistencies in existing published reports on the age dependency of T1 and its variations by sex. In our study, we found that with increasing age, native T1 increased in men and decreased in women. Dong et al^13^ report no significant age trend of native T1 in 69 healthy Chinese adults. In a study of 625 women and 606 men from the MESA (Multi-Ethnic Study of Atherosclerosis) cohort, Liu et al^12^ show increasing myocardial native T1 with age in men, but no significant age-trend in women. Similarly, in a smaller study of 75 healthy individuals, Roy et al^15^ report positive association of native T1 with increasing age in men, but no significant age trend in women. Piechnik et al^7^ report no age dependency of myocardial T1 in men and, similar to our observation, a declining trend of native T1 with increasing age in women. Rosmini et al^11^ report lower native T1 with increasing age in both men and women (using modified Look-Locker inversion recovery and ShMOLLI sequences).

The heterogeneity in age-related T1 trends reported in existing published reports may be influenced by several factors. First, there are many technical variations in image acquisition and postprocessing between studies; this makes direct between-study comparisons difficult. Second, the small sample size in many studies limits power to detect trends that require stratification by both sex and age, may not capture more complex or subtle relationships, and has the potential to generate spurious trends. Third, to appreciate age-related trends, there is need for samples which include a broad spectrum of ages; this is not always included in existing studies. Finally, the level of variation in direction of associations in existing work suggests that the pattern of age-related myocardial alteration is not a simple linear trend which is consistent across all individuals.

In the largest healthy cohort to date, our findings support the positive aging trend of T1 in men (as per Liu et al^12^ in the MESA cohort), and show a significant negative trend in women (similar to Piechnik et al^7^ toward the premenopausal stage). The sex differential age trends of native T1 in our study may indicate differences in cardiac aging patterns of men and women. They may also reflect technical factors which differentially influence measurement of T1 in men and women. Overall, it is likely that a range of biological and technical factors drive the sex differences in native T1.

### Associations with prevalent disease

Previous work shows associations of myocardial native T1 with a wide range of CVDs in patient cohorts.^3^ These are mostly small studies of selected disease subtypes with highly heterogeneous methodologies, making direct comparisons challenging. Broadly, higher native T1 appears as an indicator of disease across the published reports (except in myocardial iron overload, or significant myocardial fat, eg, Fabry disease, where the reverse is true). We examined these relationships in a population-based cohort, showing association of higher native T1 with key prevalent diseases.

We observed strong associations of higher native T1 with prevalent heart failure and nonischemic cardiomyopathies. In keeping with our findings, Dass et al,^16^ Puntmann et al,^17^ and Puyol-Anton et al^18^ all report higher native T1 in individuals with nonischemic cardiomyopathies compared to healthy controls. These observations are consistent with the global myocardial remodeling characteristic of these conditions. We also found associations of higher native T1 with prevalent IHD and MI. These associations were comparatively weaker and, although the confounder adjusted models showed statistically significant relationships, the minimally adjusted models were nonsignificant. Previous studies indicate significantly higher myocardial native T1 in focal regions of myocardial injury compared to the remote myocardium in the setting of acute MI.^19^ It is likely that predominantly focal myocardial injuries in ischemic disease are diluted within global native T1 measures.

We found higher native T1 to be linked to prevalent cardiac arrhythmias and, specifically, with prevalent AF. Previous published reports on these associations is sparse, but broadly consistent. Kato et al^20^ report higher native T1 in 50 patients with paroxysmal AF compared to 11 healthy controls. Zhao et al^21^ show higher native T1 in individuals with greater AF burden in their analysis of 108 heart failure patients. In a study of patients with nonischemic dilated cardiomyopathy, Nakamori et al^22^ suggest links between native T1 and ventricular arrhythmias, reporting higher native T1 in patients with a history of complex ventricular arrhythmia (n = 50) compared to those without (n = 57).

Our findings of positive associations between native T1 and diabetes are in keeping with previous small studies showing higher native T1 in people with diabetes compared to matched controls and consistent with the pathophysiology of diabetic cardiomyopathy.^23^^,^^24^

We observed significantly lower native T1 in participants with prevalent hypertension compared to those without hypertension. Previous studies report higher native T1 among hypertensives with left ventricular hypertrophy (LVH) compared to those without LVH.^25^^,^^26^ Thus, existing studies make comparisons among cohorts of hypertensives with different remodeling patterns, whereas we compare hypertensives vs nonhypertensives. Furthermore, given that we studied a population-based cohort, our sample includes few individuals with severe hypertensive heart disease and pathologic LVH, whereas existing work is specifically focused on hypertensives with LVH. Given these fundamental differences in study design, it is not possible to make direct comparisons with our findings. Further work is required to better understand hypertension-related variations of native T1.

### Associations with incident disease

We observed association of higher native T1 with incident AF and incident heart failure. Very few previous studies have examined the predictive value of native T1 for incident health events. In a study of 50 patients with paroxysmal AF, Kato et al^20^ report higher baseline native T1 in individuals with recurrence of AF after ablation (pulmonary vein isolation) compared to those who remained in sinus rhythm. Our findings indicate that higher native T1 is an indicator of first presentation of AF in a population setting without pre-existing AF.

### Associations with mortality

Our findings, over 3.17 ± 1.53 years of prospective follow-up, show association of native T1 with all-cause mortality, CVD mortality, and IHD mortality. Associations of native T1 with IHD mortality appeared robust, despite the previously described weaker and nonsignificant associations with prevalent and incident IHD, respectively. This may suggest that the cohort who died of IHD comprised a large proportion of individuals with severe pre-existing (prevalent) IHD phenotypes (eg, scarring and heart failure). These individuals would be expected to have more extensive myocardial abnormalities detectable by T1-mapping, which would predispose to death and drive the positive associations of T1 with IHD mortality. As individuals with severe disease phenotypes only comprise a minority of the prevalent IHD cohort, their influence on associations of T1 with prevalent IHD are diluted.

Previous studies have examined association of native T1 with incident mortality outcomes within specific disease cohorts, broadly showing significant associations of these outcomes with higher native T1. In the largest such study, Puntmann et al^27^ prospectively studied 637 patients with hypertrophic cardiomyopathy, reporting association of higher native T1 with all-cause mortality and a composite of heart failure mortality and hospitalization. Qin et al^28^ also show association of higher native T1 with adverse incident outcomes (composite of CVD death, implantable cardioverter-defibrillator placement, cardiac transplantation, myocardial infarction, heart failure, and hospitalization) in 203 patients with hypertrophic cardiomyopathy. Similarly, Garg et al^29^ report association of higher native T1 with all-cause mortality in a retrospective study of 86 patients with heart failure with preserved ejection fraction. Consistently, among 108 heart failure patients with AF, Zhao et al^21^ report higher native T1 in patients who experienced an adverse event (composite of cardiac death, stroke, and heart failure hospitalization) compared to those who did not. Furthermore, Martinez-Naharro et al^30^ observed association of higher native T1 with all-cause mortality in 227 patients with transthyretin amyloidosis, although this relationship was attenuated after adjustment for age. Our study adds important information to existing work by showing extension of these T1-mortality associations to predominantly healthy population cohorts.

Our findings show age differential pattern of T1-mortality relationships, with T1 values having stronger associations with the mortality outcomes in older ages. Furthermore, our results suggest potential nonlinearity of T1 associations with both incident disease and mortality outcomes, indicating that the magnitude of T1 associations with these outcomes was increased with higher T1 values. We advise cautious interpretation of these findings given the small number of incident events in these stratified analyses. Further studies in larger samples and with longer follow-up are required to draw more definitive conclusions.

### Clinical implications

CMR is a key research and clinical tool providing noninvasive evaluation of cardiac structure and function. Myocardial native T1 measurement allows noninvasive assessment of myocardial tissue, providing a quantitative measure of increased free water content that can characterize abnormal myocyte pathophysiology and interstitial remodeling. Myocardial native T1 instils the impact of a multitude of local and systemic cardiovascular stressors on the myocardium into a single metric. Increased native T1 has been described in a range of nonischemic and ischemic cardiomyopathies, including hypertrophic cardiomyopathy, dilated cardiomyopathy, and cardiac amyloid, whereas reduced native T1 is characteristic of Fabry disease and iron overload.^3^^,^^30^, ^31^, ^32^, ^33^, ^34^

In this study, we importantly extend existing observations in clinical cohorts to a population-based setting, showing the value of myocardial native T1 for disease discrimination and outcome prediction in a very large predominantly healthy population cohort. Our findings support high clinical utility for inclusion of myocardial native T1 measurement as a routine component of CMR studies. There is now need for validation of these relationships in other large cohorts followed by concerted efforts toward standardization of native T1-mapping techniques, which will underpin widespread clinical implementation. Furthermore, our studies lend additional weight to novel nongadolinium machine learning–based approaches that assess myocardial scar and fibrosis that are largely based on native T1, such as the recently reported virtual native enhancement method.^35^

### Study limitations

The large study sample, uniform standardized image acquisition and analysis, and linked health data in the UK Biobank provided an ideal platform for the present study. Ascertainment of mortality outcomes through data linkage with death register data is highly reliable. Similarly, incident diseases such as MI and stroke are reliably ascertained through HES records. We have shown sex-differential trends of T1 with aging among healthy participants. However, the narrow age range within our sample (44-84 years at the time of imaging) precluded examination of trends across the whole spectrum of ages; this is a particular limitation when evaluating relationships in women in whom the onset of menopause may alter the trajectory of myocardial alterations. Furthermore, we were underpowered to adequately assess sex-specific disease associations, which have relevance given major heterogeneities in CVD patterns in men and women. The purpose of this study is to provide a broad overview of clinical associations of native T1; it is possible that the observed relationships may have specificity within disease subtypes, and future work dedicated to elucidating these more granular associations is required. As outcomes accrue in the UK Biobank, it will be possible to examine more disease-specific associations and to consider relationships in sex-stratified models. The reported findings have been derived from a fraction of the planned UK Biobank cohort, pending confirmation on the complete total 100,000 studies, including possible use of independent methods for automatic segmentation (eg, Puyol-Anton et al^18^). The UK Biobank CMR protocol does not include contrast administration; as such, broader comparison of our findings with tissue characterization methods requiring contrast, such as late gadolinium enhancement or extracellular volume, was not possible. Another key question for future work is to determine whether native T1 measurement provides incremental risk information over existing standard morphological CMR metrics. Finally, these modern in vivo T1 measurements are subject to multiple confounders and great care needs to be exercised when applying the observations to other techniques until the ongoing standardization effort is successfully completed.^36^

## Conclusions

In this large population cohort of 42,308 UK Biobank participants, we show significant associations of intrinsic properties of the living myocardial tissue, as measured by myocardial native T1, with a wide range of prevalent and incident CVDs. Critically, we show novel associations of myocardial T1 with all-cause mortality, CVD mortality, and IHD mortality. Our findings support wider use of myocardial native T1 in routine clinical practice.

Perspectives **COMPETENCY IN MEDICAL KNOWLEDGE:** In this analysis of 42,308 UK Biobank participants, we show associations of higher native T1 with a range of cardiovascular risk factors and diseases. Importantly, we show the value of native T1 as a reliable indicator of incident cardiovascular disease and mortality outcomes in a predominantly healthy population-based cohort.

**TRANSLATIONAL OUTLOOK:** Our findings significantly extend the remit of native T1 outside of very specific diseases contexts (reported in previous work) into wider populations. Our results support clinical utility for wider use of native T1 sequences and their inclusion in routine CMR protocols. Given this hugely expanded remit, there is now urgent need for standardization of native T1 techniques to establish universal reference distributions and to ensure global comparability.

## Funding Support and Author Disclosures

This project was enabled through access to the Medical Research Council (MRC) eMedLab Medical Bioinformatics infrastructure (www.mrc.ac.uk; MR/L016311/1). This work was supported by Health Data Research UK, an initiative funded by UK Research and Innovation, Department of Health and Social Care (England) and the devolved administrations, and leading medical research charities. This study was conducted using the UK Biobank resource under access application 2964. The British Heart Foundation (BHF) funded the manual image analysis underpinning the creation of a cardiovascular magnetic resonance imaging reference standard for the UK Biobank imaging resource in 5000 scans (www.bhf.org.uk; PG/14/89/31194). Dr Raisi-Estabragh has received grants from the BHF Clinical Research Training Fellowship (No. FS/17/81/33318) and recognizes the National Institute for Health Research (NIHR) Integrated Academic Training programme which supports her Academic Clinical Lectureship post. Ms McCracken and Drs Neubauer, Ferreira, and Piechnik have received grants from the Oxford NIHR Biomedical Research Centre and the Oxford BHF Centre of Research Excellence. Dr Harvey has received grants from MRC (MC_UU_12011/1) and NIHR Southampton Biomedical Research Centre. Drs Munroe and Petersen have received grants from the National Institute for Health Research (NIHR) Biomedical Research Centre at Barts. Dr Petersen has received funding from the European Union’s Horizon 2020 research and innovation program under grant agreement No 825903 (euCanSHare project), and the “SmartHeart” EPSRC program grant (www.nihr.ac.uk; EP/P001009/1); and has received consulting fees from Cardiovascular Imaging Inc, Calgary, Alberta, Canada. All other authors have reported that they have no relationships relevant to the contents of this paper to disclose.

## Appendix

Supplemental Tables 1-7 and Suplemental Figure 1

## References

[1] Taylor A.J., Salerno M., Dharmakumar R., Jerosch-Herold M. (2016). T1 Mapping. J Am Coll Cardiol Img.

[2] Kramer C.M., Chandrashekhar Y., Narula J. (2013). T1 mapping by CMR in cardiomyopathy: a noninvasive myocardial biopsy?. J Am Coll Cardiol Img.

[3] Messroghli D.R., Moon J.C., Ferreira V.M. (2017). Clinical recommendations for cardiovascular magnetic resonance mapping of T1, T2, T2∗ and extracellular volume: a consensus statement by the Society for Cardiovascular Magnetic Resonance (SCMR) endorsed by the European Association for Cardiovascular Imaging. J Cardiovasc Magn Reson.

[4] Raisi-Estabragh Z., Harvey N.C., Neubauer S., Petersen S.E. (2021). Cardiovascular magnetic resonance imaging in the UK Biobank: a major international health research resource. Eur Heart J Cardiovasc Imaging.

[5] UK Biobank Coordinating Centre UK Biobank: Protocol for a large-scale prospective epidemiological resource. https://www.ukbiobank.ac.uk/media/gnkeyh2q/study-rationale.pdf.

[6] Petersen S.E., Matthews P.M., Francis J.M. (2015). UK Biobank’s cardiovascular magnetic resonance protocol. J Cardiovasc Magn Reson.

[7] Piechnik S.K., Ferreira V.M., Lewandowski A.J. (2013). Normal variation of magnetic resonance T1 relaxation times in the human population at 1.5 T using ShMOLLI. J Cardiovasc Magn Reson.

[8] Hann E., Popescu I.A., Zhang Q. (2021). Deep neural network ensemble for on-the-fly quality control-driven segmentation of cardiac MRI T1 mapping. Med Image Anal.

[9] Raisi-Estabragh Z., McCracken C., Condurache D. (2021). Left atrial structure and function are associated with cardiovascular outcomes independent of left ventricular measures: a UK Biobank CMR study. Eur Heart J Cardiovasc Imaging.

[10] Benjamini Y., Hochberg Y. (1995). Controlling the False discovery rate: a practical and powerful approach to multiple testing. Source J R Stat Soc Ser B.

[11] Rosmini S., Bulluck H., Captur G. (2018). Myocardial native T1 and extracellular volume with healthy ageing and gender. Eur Heart J Cardiovasc Imaging.

[12] Liu C.Y., Liu Y.C., Wu C. (2013). Evaluation of age-related interstitial myocardial fibrosis with cardiac magnetic resonance contrast-enhanced T1 mapping. J Am Coll Cardiol.

[13] Dong Y., Yang D., Han Y. (2018). Age and gender impact the measurement of myocardial interstitial fibrosis in a healthy adult Chinese population: a cardiac magnetic resonance study. Front Physiol.

[14] Rauhalammi S.M.O., Mangion K., Barrientos P.H. (2016). Native myocardial longitudinal (T1) relaxation time: regional, age, and sex associations in the healthy adult heart. J Magn Reson Imaging.

[15] Roy C., Slimani A., De Meester C. (2017). Age and sex corrected normal reference values of T1, T2 T2∗ and ECV in healthy subjects at 3T CMR. J Cardiovasc Magn Reson.

[16] Dass S., Suttie J.J., Piechnik S.K. (2012). Myocardial tissue characterization using magnetic resonance noncontrast T1 mapping in hypertrophic and dilated cardiomyopathy. Circ Cardiovasc Imaging.

[17] Puntmann V.O., Voigt T., Chen Z. (2013). Native T1 mapping in differentiation of normal myocardium from diffuse disease in hypertrophic and dilated cardiomyopathy. J Am Coll Cardiol Img.

[18] Puyol-Antón E., Ruijsink B., Baumgartner C.F. (2020). Automated quantification of myocardial tissue characteristics from native T1 mapping using neural networks with uncertainty-based quality-control. J Cardiovasc Magn Reson.

[19] Dall’Armellina E., Piechnik S.K., Ferreira V.M. (2012). Cardiovascular magnetic resonance by non contrast T1-mapping allows assessment of severity of injury in acute myocardial infarction. J Cardiovasc Magn Reson.

[20] Kato S., Foppa M., Roujol S. (2016). Left ventricular native T1 time and the risk of atrial fibrillation recurrence after pulmonary vein isolation in patients with paroxysmal atrial fibrillation. Int J Cardiol.

[21] Zhao L., Li S., Ma X. (2019). Prognostic significance of left ventricular fibrosis assessed by T1 mapping in patients with atrial fibrillation and heart failure. Sci Rep.

[22] Nakamori S., Bui A.H., Jang J. (2018). Increased myocardial native T 1 relaxation time in patients with nonischemic dilated cardiomyopathy with complex ventricular arrhythmia. J Magn Reson Imaging.

[23] Lam B., Stromp T.A., Hui Z., Vandsburger M. (2019). Myocardial native-T1 times are elevated as a function of hypertrophy, HbA1c, and heart rate in diabetic adults without diffuse fibrosis. Magn Reson Imaging.

[24] Vasanji Z., Sigal R.J., Eves N.D. (2017). Increased left ventricular extracellular volume and enhanced twist function in type 1 diabetic individuals. J Appl Physiol.

[25] Rodrigues J.C.L., Amadu A.M., Dastidar A.G. (2016). Comprehensive characterisation of hypertensive heart disease left ventricular phenotypes. Heart.

[26] Treibel T.A., Zemrak F., Sado D.M. (2015). Extracellular volume quantification in isolated hypertension—changes at the detectable limits?. J Cardiovasc Magn Reson.

[27] Puntmann V.O., Carr-White G., Jabbour A. (2016). T1-mapping and outcome in nonischemic cardiomyopathy all-cause mortality and heart failure. J Am Coll Cardiol Img.

[28] Qin L., Min J., Chen C. (2021). Incremental values of T1 mapping in the prediction of sudden cardiac death risk in hypertrophic cardiomyopathy: a comparison with two guidelines. Front Cardiovasc Med.

[29] Garg P., Assadi H., Jones R. (2021). Left ventricular fibrosis and hypertrophy are associated with mortality in heart failure with preserved ejection fraction. Sci Rep.

[30] Martinez-Naharro A., Kotecha T., Norrington K. (2019). Native T1 and extracellular volume in transthyretin amyloidosis. J Am Coll Cardiol Img.

[31] Arcari L., Hinojar R., Engel J. (2020). Native T1 and T2 provide distinctive signatures in hypertrophic cardiac conditions — comparison of uremic, hypertensive and hypertrophic cardiomyopathy. Int J Cardiol.

[32] Yanagisawa F., Amano Y., Tachi M., Inui K., Asai K., Kumita S. (2019). Non–contrast-enhanced T1 mapping of dilated cardiomyopathy: comparison between native T1 values and late gadolinium enhancement. Magn Reson Med Sci.

[33] Deborde E., Dubourg B., Bejar S. (2020). Differentiation between Fabry disease and hypertrophic cardiomyopathy with cardiac T1 mapping. Diagn Interv Imaging.

[34] Meloni A., Martini N., Positano V. (2021). Myocardial iron overload by cardiovascular magnetic resonance native segmental T1 mapping: a sensitive approach that correlates with cardiac complications. J Cardiovasc Magn Reson.

[35] Zhang Q., Burrage M.K., Lukaschuk E. (2021). Toward replacing late gadolinium enhancement with artificial intelligence virtual native enhancement for gadolinium-free cardiovascular magnetic resonance tissue characterization in hypertrophic cardiomyopathy. Circulation.

[36] Zhang Q., Werys K., Popescu I.A. (2021). Quality assurance of quantitative cardiac T1-mapping in multicenter clinical trials—a T1 phantom program from the hypertrophic cardiomyopathy registry ( HCMR ) study. Int J Cardiol.

