# Supplementary material for: Incident Clinical and Mortality Associations of Myocardial Native T1 in the UK Biobank
Source: JACC Cardiovasc Imaging. 2023 Apr;16(4):450–60. doi: 10.1016/j.jcmg.2022.06.011 (PMC10102720; doi:10.1016/j.jcmg.2022.06.011)
Supplement: Supplemental Tables 1-7 and Suplemental Figure 1 [file mmc1.docx]

**Supplemental Figure 1. Summary of approach to selection of participants**

Native T1 available

n = 42,894

Excluded:

Dice score <0.7 (n = 586)

Excluded:

any CVD (n = 4,885)

any VRF (n = 18,126)

Analysable native T1 n = 42,308

**Healthy subset**

n = 19,297

**Clinical associations**

n = 42,308

VRF: vascular risk factors. CVD: cardiovascular disease. VRFs included: diabetes, hypertension, high cholesterol, or smoking.

**Supplemental Table 1. Disease definitions**

| **Source** | **UKB Field ID / Code** | **Description** |
| --- | --- | --- |
| **Alzheimer’s / dementia** | | |
| Self-report | 20002 | dementia/Alzheimer’s/cognitive impairment |
| ICD9 | 290 | Senile and presenile organic psychotic conditions |
|  | 331 | Other cerebral degenerations |
| ICD10 | F00.0 | Dementia in Alzheimer's disease with early onset |
|  | F00.1 | Dementia in Alzheimer's disease with late onset |
|  | F00.2 | Dementia in Alzheimer's disease, atypical or mixed type |
|  | F00.9 | Dementia in Alzheimer's disease, unspecified |
|  | F01 | Vascular dementia |
|  | F02 | Dementia in other diseases classified elsewhere |
|  | F03 | Unspecified dementia |
|  | F05.1 | Delirium superimposed on dementia |
|  | G30.0 | Alzheimer's disease with early onset |
|  | G30.1 | Alzheimer's disease with late onset |
|  | G30.8 | Other Alzheimer's disease |
|  | G30.9 | Alzheimer's disease, unspecified |
|  | G31 | Other degenerative diseases of nervous system, not elsewhere classified |
|  | I67.3 | Progressive vascular leukoencephalopathy |
| First occurrences | 130836 | dementia in Alzheimer’s disease |
|  | 130838 | vascular dementia |
|  | 130840 | dementia in other diseases classified elsewhere |
|  | 130842 | unspecified dementia |
|  | 131036 | Alzheimer’s disease |
|  | 131038 | other degenerative diseases of nervous system, not elsewhere classified |
| Algorithm | 42022 | Date of vascular dementia report |
|  | 42018 | Date of all cause dementia report |
|  | 42024 | Date of frontotemporal dementia report |
|  | 42020 | Date of Alzheimer’s disease report |
| **Benign neoplasm of brain, meninges and other parts of the CNS** | | |
| Self-report | 20002 | benign neuroma |
|  | 20002 | meningioma / benign meningeal tumour |
| ICD10 | D32 | Benign neoplasm of meninges |
|  | D33 | Benign neoplasm of brain and other parts of central nervous system |
| Date of cancer | 40005 | Match to field 40006 |
| Cancer register | 40006: D32 | Benign neoplasm of meninges |
|  | 40006: D33 | Benign neoplasm of brain and other parts of central nervous system |
| **Brain abscess/intracranial abscess** | | |
| Self-report | 20002 | brain abscess/intracranial abscess |
|  | 20002 | spinal abscess |
| ICD10 | G06 | Intracranial and intraspinal abscess and granuloma |
|  | G07 | Intracranial and intraspinal abscess and granuloma in diseases classified elsewhere |
|  | G08 | Intracranial and intraspinal phlebitis and thrombophlebitis |
|  | G09 | Sequelae of inflammatory diseases of CNS |
| First occurrences | 131004 | intracranial and intraspinal abscess and granuloma |
|  | 131006 | intracranial and intraspinal abscess and granuloma in diseases classified elsewhere |
|  | 131008 | intracranial and intraspinal phlebitis and thrombophlebitis |
|  | 131010 | sequelae of inflammatory diseases of central nervous system |
| **Cerebral palsy** |  |  |
| Self-report | 20002 | cerebral palsy |
| ICD10 | G80 | Cerebral palsy |
| First occurrences | 131100 | cerebral palsy |
| **Cerebrovascular diseases** | | |
| ICD10 | I65 | Occlusion and stenosis of precerebral arteries, not resulting in cerebral infarction |
|  | I66 | Occlusion and stenosis of cerebral arteries, not resulting in cerebral infarct |
|  | I67 | Other cerebrovascular diseases |
|  | I68 | Cerebrovascular disorders in diseases classified elsewhere |
| First occurrences | 131370 | Date I65 first reported (occlusion and stenosis of precerebral arteries, not resulting in cerebral infarction) |
|  | 131372 | Date I66 first reported (occlusion and stenosis of cerebral arteries, not resulting in cerebral infarction) |
|  | 131374 | Date I67 first reported (other cerebrovascular diseases) |
|  | 131376 | Date I68 first reported (cerebrovascular disorders in diseases classified elsewhere) |
| **Epilepsy** |  |  |
| Self-report | 20002 | epilepsy |
| ICD10 | G40 | Epilepsy |
|  | G41 | Status epilepticus |
| First occurrences | 131048 | Epilepsy |
|  | 131050 | Status epilepticus |
| **Inflammatory diseases of the CNS - Meningitis** | | |
| Self-report | 20002 | meningitis |
|  | 20002 | infection of nervous system |
| ICD10 | G00 | Bacterial meningitis, not elsewhere classified |
|  | G01 | Meningitis in bacterial diseases classified elsewhere |
|  | G03 | Meningitis due to other and unspecified causes |
| First occurrences | 130992 | bacterial meningitis, not elsewhere classified |
|  | 130994 | meningitis in bacterial diseases classified elsewhere |
|  | 130998 | meningitis due to other and unspecified causes |
| Self-report | 20002 | encephalitis |
| ICD10 | G04.0 | Acute disseminated encephalitis |
|  | G04.2 | Bacterial meningoencephalitis and meningomyelitis, not elsewhere classified |
|  | G04.8 | Other encephalitis, myelitis and encephalomyelitis |
|  | G04.9 | Encephalitis, myelitis and encephalomyelitis, unspecified |
|  | G05 | Encephalitis, myelitis and encephalomyelitis in diseases classified elsewhere |
| First occurrences | 131000 | Encephalitis, myelitis and encephalomyelitis |
|  | 131002 | Encephalitis, myelitis and encephalomyelitis in diseases classified elsewhere |
| **Malignant neoplasm of brain** | | |
| Self-report | 20001 | Brain cancer / primary malignant brain tumour |
|  | 20001 | Spinal cord or cranial nerve cancer |
| ICD10 | C71.0 | Cerebrum, except lobes and ventricles |
|  | C71.1 | Frontal lobe |
|  | C71.2 | Temporal lobe |
|  | C71.3 | Parietal lobe |
|  | C71.4 | Occipital lobe |
|  | C71.5 | Cerebral ventricle |
|  | C71.6 | Cerebellum |
|  | C71.7 | Brain stem |
|  | C71.8 | Overlapping lesion of brain |
|  | C71.9 | Brain, unspecified |
|  | C72.0 | Spinal cord |
| Date of cancer | 40005 | Match to field 40006 |
| Cancer register | 40006: C71 | Malignant neoplasm of brain |
|  | 40006: C72.0 | Spinal cord |
| **Motor neuron disease (and other spinal muscular atrophies)** | | |
| Self-report | 20002 | Motor neurone disease |
| ICD10 | G12 | Motor neuron disease and other spinal muscular atrophies |
| First occurrences | 131016 | Spinal muscular atrophy and related syndromes |
| Algorithm | 42028 | Date of motor neurone disease report |
| **Multiple Sclerosis** |  |  |
| Self-report | 20002 | Multiple sclerosis |
| ICD10 | G35 | Multiple sclerosis |
| First occurrences | 131042 | Multiple sclerosis |
| **Myasthenia gravis and other myoneural disorders** | | |
| Self-report | 20002 | Myasthenia gravis |
| ICD10 | G70 | Myasthenia gravis and other myoneural disorders |
| First occurrences | 131092 | Myasthenia gravis and other myoneural disorders |
| **Neurological injury/trauma** | | |
| Self-report | 20002 | Neurological injury/trauma |
| ICD10 | S06 | Intracranial injury |
| **Organic, including symptomatic, mental disorders** | | |
| ICD10 | F06 | Other mental disorders due to brain damage and dysfunction and to physical disease |
|  | F07 | Personality and behavioural disorders due to brain disease, damage and dysfunction |
|  | F09 | Unspecified organic or symptomatic mental disorder |
|  | F70 | Mild mental retardation |
|  | F71 | Moderate mental retardation |
|  | F72 | Severe mental retardation |
|  | F73 | Profound mental retardation |
|  | F78 | Other mental retardation |
|  | F79 | Unspecified mental retardation |
| First occurrences | 130848 | Other mental disorders due to brain damage and dysfunction and to physical disease |
|  | 130850 | Personality and behavioural disorders due to brain disease, damage and dysfunction |
|  | 130852 | Unspecified organic or symptomatic mental disorder |
|  | 130950 | Mild mental retardation |
|  | 130952 | Moderate mental retardation |
|  | 130954 | Severe mental retardation |
|  | 130958 | Other mental retardation |
|  | 130960 | Unspecified mental retardation |
| **Other degenerative diseases of the nervous system** | | |
| Self-report | 20002 | Chronic/degenerative neurological problem |
| ICD10 | F04 | Organic amnesic syndrome, not induced by alcohol and other psychoactive substances |
|  | G23 | Other degenerative diseases of basal ganglia |
|  | G24.0 | Drug-induced dystonia |
|  | G24.1 | Idiopathic familial dystonia |
|  | G24.2 | Idiopathic nonfamilial dystonia |
|  | G24.8 | Other dystonia |
|  | G24.9 | Dystonia, unspecified |
|  | G25.3 | Myoclonus |
|  | G25.4 | Drug-induced chorea |
|  | G25.5 | Other chorea |
|  | G25.8 | Other specified extrapyramidal and movement disorders |
|  | G25.9 | Extrapyramidal and movement disorder, unspecified |
|  | G32 | Other degenerative disorders of nervous system in diseases classified elsewhere |
| First occurrences | 130844 | Organic amnesic syndrome, not induced by alcohol and other psychoactive substances |
|  | 131028 | Other degenerative diseases of basal ganglia |
|  | 131040 | Other degenerative disorders of nervous system in diseases classified elsewhere |
| Algorithm | 42034 | Date of progressive supranuclear palsy report |
|  | 42036 | Date of multiple system atrophy report |
| **Other demyelinating diseases of CNS** | | |
| Self-report | 20002 | Other demyelinating disease (not multiple sclerosis) |
| ICD10 | G36 | Other acute disseminated demyelination |
|  | G37 | Other demyelinating diseases of central nervous system |
| First occurrences | 131044 | Other acute disseminated demyelination |
|  | 131046 | Other demyelinating diseases of central nervous system |
| **Other disorders of the nervous system** | | |
| Self-report | 20002 | Spina bifida |
|  | 20002 | Spinal cord disorder |
| ICD10 | Q00 | Anencephaly and similar malformations |
|  | Q01 | Encephalocele |
|  | Q02 | Microcephaly |
|  | Q03 | Congenital hydrocephalus |
|  | Q04 | Other congenital malformations of brain |
|  | Q05 | Spina bifida |
|  | Q06 | Other congenital malformations of spinal cord |
|  | Q07 | Other congenital malformations of nervous system |
|  | G91 | Hydrocephalus |
|  | G92 | Toxic encephalopathy |
|  | G93.1 | Anoxic brain damage, not elsewhere classified |
|  | G93.2 | Benign intracranial hypertension |
|  | G93.4 | Encephalopathy, unspecified |
|  | G93.5 | Compression of brain |
|  | G93.6 | Cerebral oedema |
|  | G94 | Other disorders of brain in diseases classified elsewhere |
|  | G95 | Other diseases of spinal cord |
| First occurrences | 132432 | Anencephaly and similar malformations |
|  | 132434 | Encephalocele |
|  | 132436 | Microcephaly |
|  | 132438 | Congenital hydrocephalus |
|  | 132440 | Other congenital malformations of brain |
|  | 132442 | Spina bifida |
|  | 132444 | Other congenital malformations of spinal cord |
|  | 132446 | Other congenital malformations of nervous system |
|  | 131110 | Hydrocephalus |
|  | 131112 | Toxic encephalopathy |
|  | 131114 | Other disorders of brain |
|  | 131116 | Other disorders of brain in diseases classified elsewhere |
|  | 131118 | Other diseases of spinal cord |
| **Other mental and behavioural disorders** | | |
| Self-report | 20002 | Schizophrenia |
|  | 20002 | Mania/bipolar disorder/manic depression |
|  | 20002 | Obsessive compulsive disorder (OCD) |
|  | 20002 | Anorexia/bulimia/other eating disorder |
| ICD10 | F20 | Schizophrenia |
|  | F21 | Schizotypal disorder |
|  | F30 | Manic episode |
|  | F31 | Bipolar affective disorder |
|  | F42 | Obsessive-compulsive disorder |
|  | F50 | Eating disorders |
| First occurrences | 130874 | Schizophrenia |
|  | 130876 | Schizotypal disorder |
|  | 130890 | Manic episode |
|  | 130892 | Bipolar affective disorder |
|  | 130908 | Obsessive-compulsive disorder |
|  | 130918 | Eating disorders |
| **Parkinson’s disease** | |  |
| Self-report | 20002 | Parkinson’s disease |
| ICD9 | 332 | Parkinson's disease |
| ICD10 | G22 | Parkinsonism in diseases classified elsewhere |
|  | G20 | Parkinson’s disease |
|  | G21 | Secondary Parkinsonism |
| First occurrences | 131022 | Parkinson’s disease |
|  | 131024 | Secondary parkinsonism |
|  | 131026 | Parkinsonism in diseases classified elsewhere |
| Algorithm | 42030 | Date of all cause parkinsonism report |
|  | 42032 | Date of Parkinson’s disease report |
| **Subarachnoid hemorrhage** | | |
| Self-report | 20002 | Subarachnoid hemorrhage |
| ICD9 | 430 | Subarachnoid hemorrhage |
| ICD10 | I60 | Subarachnoid hemorrhage |
| First occurrences | 131360 | Subarachnoid hemorrhage |
| Algorithm | 42012 | Date of subarachnoid hemorrhage (should be covered by 42006) |
| **Systemic atrophies primarily affecting the CNS** | | |
| ICD10 | G10 | Huntington's disease |
|  | G11 | Hereditary ataxia |
|  | G13 | Systemic atrophies primarily affecting central nervous system in diseases Classified elsewhere |
| First occurrences | 131012 | Huntington’s disease |
|  | 131014 | Hereditary ataxia |
|  | 131018 | Systemic atrophies primarily affecting central nervous system in diseases classified elsewhere |
| **Transient ischaemic attack (TIA)** | | |
| Self-report | 20002 | Transient ischaemic attack (TIA) |
| ICD9 | 435 | Transient cerebral ischaemia |
| ICD10 | G45 | Transient cerebral ischaemic attacks and related syndromes |
| First occurrences | 131056 | Transient cerebral ischaemic attacks and related syndromes |
| **Stroke** |  |  |
| Self-report | 20002 | Stroke |
|  | 20002 | Ischaemic stroke |
|  | 20002 | Brain hemorrhage |
| ICD9 | 431 | Intracerebral hemorrhage |
|  | 432 | Other and unspecified intracranial hemorrhage |
| ICD10 | I64 | Stroke, not specified as hemorrhage or infarction |
|  | I63 | Cerebral infarction |
|  | I61 | Intracerebral hemorrhage |
|  | I62 | Other nontraumatic intracranial hemorrhage |
| First occurrences | 131368 | Date I64 first reported (stroke, not specified as hemorrhage or infarction) |
|  | 131366 | Cerebral infarction |
|  | 131362 | Intracerebral hemorrhage |
|  | 131364 | Other nontraumatic intracranial hemorrhage |
| Diagnosed by doctor | 4056 | Age stroke diagnosed |
|  | 6150: 3 | Stroke |
| Algorithm | 42006 | Date of stroke |
|  | 42008 | Date of ischaemic stroke |
|  | 42010 | Date of intracerebral hemorrhage |
| **Cardiac arrhythmia** | | |
| Self-report | 20002 | Sick sinus syndrome |
|  | 20002 | SVT / supraventricular tachycardia |
|  | 20002 | Atrial flutter |
|  | 20002 | Heart arrhythmia |
|  | 20002 | Irregular heart beat |
| ICD10 | I44.1 | Atrioventricular block, second degree |
|  | I44.2 | Atrioventricular block, complete |
|  | I45.3 | Trifascicular block |
|  | I45.6 | Preexcitation syndrome |
|  | I46.0 | Cardiac arrest with successful resuscitation |
|  | I46.1 | Sudden cardiac death, so described |
|  | I46.9 | Cardiac arrest, unspecified |
|  | I47.0 | Re-entry ventricular arrhythmia |
|  | I47.1 | Supraventricular tachycardia |
|  | I47.2 | Ventricular tachycardia |
|  | I47.9 | Paroxysmal tachycardia, unspecified |
|  | I48.3 | Typical atrial flutter |
|  | I48.4 | Atypical atrial flutter |
|  | I49.0 | Ventricular fibrillation and flutter |
|  | I49.5 | Sick sinus syndrome |
| First occurrences | 131346 | Cardiac arrest |
|  | 131348 | Paroxysmal tachycardia |
|  | 131350 | Atrial fibrillation and flutter |
| **Cardiac arrhythmia (Atrial fibrillation)** | | |
| Self-report | 20002 | Atrial fibrillation |
| ICD10 | I48.0 | Paroxysmal atrial fibrillation |
|  | I48.1 | Persistent atrial fibrillation |
|  | I48.2 | Chronic atrial fibrillation |
|  | I48.9 | Atrial fibrillation and atrial flutter, unspecified |
| **Heart failure (unspecified etiology)** | | |
| Self-report | 20002 | Heart failure/pulmonary oedema |
| ICD10 | I50.0 | Congestive heart failure |
|  | I50.1 | Left ventricular failure |
|  | I50.9 | Heart failure, unspecified |
| First occurrences | 131354 | Heart failure |
| **Ischaemic heart disease** | | |
| Self-report | 20002 | Angina |
| ICD10 | I20 | Angina pectoris |
|  | I24 | Other acute ischaemic heart diseases |
|  | I25 | Chronic ischaemic heart disease |
| First occurrences | 131296 | Angina pectoris |
|  | 131304 | Other acute ischaemic heart diseases |
|  | 131306 | Chronic ischaemic heart disease |
| Diagnosed by doctor | 3627 | Age angina diagnosed |
|  | 6150: 2 | Angina |
| **Ischaemic heart disease (Myocardial infarction)** | | |
| Self-report | 20002 | Heart attack/myocardial infarction |
| ICD9 | 410 | Acute myocardial infarction |
|  | 411 | Other acute and subacute forms of ischaemic heart disease |
|  | 412 | Old myocardial infarction |
| ICD10 | I21 | Acute myocardial infarction |
|  | I22 | Subsequent myocardial infarction |
|  | I23 | Certain current complications following acute myocardial infarction |
| First occurrences | 131298 | Acute myocardial infarction |
|  | 131300 | Subsequent myocardial infarction |
|  | 131302 | Certain current complications following acute myocardial infarction |
| Diagnosed by doctor | 3894 | Age heart attack diagnosed |
|  | 6150: 1 | Heart attack |
| Algorithm | 42000 | Date of myocardial infarction |

| **Non-ischaemic cardiomyopathies** | | |
| --- | --- | --- |
| Self-report | 20002 | Cardiomyopathy |
|  | 20002 | Hypertrophic cardiomyopathy (HCM / HOCM) |
| ICD10 | I42 | Cardiomyopathy |
|  | I43 | Cardiomyopathy in diseases classified elsewhere |
|  | I11 | Hypertensive heart disease |
|  | I13 | Hypertensive heart and renal disease |
| First occurrences | 131338 | Cardiomyopathy |
|  | 131340 | Cardiomyopathy in diseases classified elsewhere |
|  | 131288 | Hypertensive heart disease |
|  | 131292 | Hypertensive heart and renal disease |
| **Valvular heart disease** | | |
| Self-report | 20002 | Mitral stenosis |
|  | 20002 | Mitral valve disease |
|  | 20002 | Heart valve problem/heart murmur |
|  | 20002 | Mitral regurgitation / incompetence |
|  | 20002 | Aortic valve disease |
|  | 20002 | Aortic stenosis |
|  | 20002 | Aortic regurgitation / incompetence |
| ICD10 | I34.0 | Mitral (valve) insufficiency |
|  | I34.2 | Non-rheumatic mitral (valve) stenosis |
|  | I34.8 | Other nonrheumatic mitral valve disorders |
|  | I34.9 | Non-rheumatic mitral valve disorder, unspecified |
|  | I35 | Non-rheumatic aortic valve disorders |
|  | I36 | Non-rheumatic tricuspid valve disorders |
|  | I37 | Pulmonary valve disorders |
|  | I38 | Endocarditis, valve unspecified |
|  | I39.0 | Mitral valve disorders in diseases classified elsewhere |
|  | I39.1 | Aortic valve disorders in diseases classified elsewhere |
|  | I39.3 | Pulmonary valve disorders in diseases classified elsewhere |
|  | I39.4 | Multiple valve disorders in diseases classified elsewhere |
|  | I39.8 | Endocarditis, valve unspecified, in diseases classified elsewhere |
|  | I05 | Rheumatic mitral valve diseases |
|  | I06 | Rheumatic aortic valve diseases |
|  | I07 | Rheumatic tricuspid valve diseases |
|  | I08 | Multiple valve diseases |
| First occurrences | 131322 | Non-rheumatic mitral valve disorders |
|  | 131324 | Non-rheumatic aortic valve disorders |
|  | 131326 | Non-rheumatic tricuspid valve disorders |
|  | 131328 | Pulmonary valve disorders |
|  | 131330 | Endocarditis, valve unspecified |
|  | 131332 | Endocarditis and heart valve disorders in diseases classified elsewhere |
|  | 131276 | Rheumatic mitral valve diseases |
|  | 131278 | Rheumatic aortic valve diseases |
|  | 131280 | Rheumatic tricuspid valve diseases |
|  | 131282 | Multiple valve diseases |
| **Diabetes** |  |  |
| Self-report | 20002 | Diabetes |
|  | 20002 | Type 1 diabetes |
|  | 20002 | Type 2 diabetes |
| Medications | 6177, 6153: 3 | Insulin |
| ICD9 | 250 | Diabetes mellitus |
| ICD10 | E10 | Type 1 diabetes mellitus |
|  | E11 | Type 2 diabetes mellitus |
|  | E13 | Other specified diabetes mellitus |
|  | E14 | Unspecified diabetes mellitus |
|  | G590 | Diabetic mononeuropathy |
|  | G632 | Diabetic polyneuropathy |
|  | H280 | Diabetic cataract |
|  | H360 | Diabetic retinopathy |
|  | M142 | Diabetic arthropathy |
|  | N083 | Glomerular disorders in diabetes mellitus |
|  | O240 | Diabetes mellitus in pregnancy: Pre-existing type 1 diabetes mellitus |
|  | O241 | Diabetes mellitus in pregnancy: Pre-existing type 2 diabetes mellitus |
|  | O243 | Diabetes mellitus in pregnancy: Pre-existing diabetes mellitus, unspecified |
|  | O244 | Diabetes mellitus arising in pregnancy |
|  | O249 | Diabetes mellitus in pregnancy, unspecified |
|  | Y423 | Insulin and oral hypoglycemic [antidiabetic] drugs |
| First occurrences | 130706 | Date E10 first reported (insulin-dependent diabetes mellitus) |
|  | 130708 | Date E11 first reported (non-insulin-dependent diabetes mellitus) |
|  | 130712 | Date E13 first reported (other specified diabetes mellitus) |
|  | 130714 | Date E14 first reported (unspecified diabetes mellitus) |
| Diagnosed by doctor | 2443 | Diabetes diagnosed by doctor |
|  | 2976 | Age diabetes diagnosed by doctor |
| Biochemistry | 30750 | Glycated hemoglobin (HbA1c) >48 mmol/L |
| **High cholesterol** |  |  |
| Self-report | 20002 | High cholesterol |
| Medications | 6177, 6153: 1 | Cholesterol lowering medication |
| ICD10 | E780 | Pure hypercholesterolemia |
|  | E782 | Mixed hyperlipidemia |
|  | E783 | Hyperchylomicronaemia |
|  | E784 | Other hyperlipidemia |
|  | E785 | Hyperlipidemia, unspecified |
| First occurrences | 130814 | Date E78 first reported (disorders of lipoprotein metabolism and other lipidemias) |
| Biochemistry | 30690 | Cholesterol >7mmol/L |
| **Hypertension** |  |  |
| Self-report | 20002 | Essential hypertension |
|  | 20002 | Hypertension |
| Medications | 6177, 6153: 2 | Blood pressure medication |
| ICD10 | I10 | Essential (primary) hypertension |
| First occurrences | 131286 | Date I10 first reported (essential (primary) hypertension) |
| Diagnosed by doctor | 2966 | Age high blood pressure diagnosed |
|  | 6150: 4 | High blood pressure |

Where a 3-digit ICD10 code is given, this includes all subsections (e.g., I21 includes I21.0, I21.1 etc.)

**Supplemental Table 2. The association of age with myocardial native T1 in healthy men and women**

| Sample | Standardised beta | 95% CI | p-value | N |
| --- | --- | --- | --- | --- |
| Women | -0.33* | [-0.41, -0.24] | <0.0001 | 11,479 |
| Men | 0.48* | [0.39, 0.57] | <0.0001 | 7,818 |

Linear regression models with myocardial native T1 set as the outcome (response variable) and age as the exposure of interest, separately in men and women. Results are standard deviation change in myocardial native T1 per 1 standard deviation increase in age (7.7 years) in the healthy cohort.

**Supplemental Table 3. Assessment of significant interaction of age and sex with T1 in the relationships with incident events**

|  | T1 x age | T1 x sex |
| --- | --- | --- |
| Incident cardiovascular disease (any) | 0.0334 | 0.7244 |
| Incident atrial fibrillation | 0.3551 | 0.7699 |
| Incident heart failure | 0.7385 | 0.1438 |
| Incident stroke | 0.3062 | 0.4400 |
| Incident myocardial infarction | 0.0413 | 0.9447 |
| Incident ischaemic heart disease | 0.4759 | 0.3203 |
| All-cause mortality | **0.0026** | 0.0894 |
| Cardiovascular disease mortality | **2.04 x 10^-4^** | 0.1506 |
| Ischaemic heart disease mortality | **2.41 x 10^-5^** | 0.9055 |

Results are p-values corresponding to the interaction terms T1 x age and T1 x sex- when added to our main models with incident disease and mortality events as the outcome.

**Supplemental Table 4. The associations of native T1 with mortality outcomes stratified by median age**

|  |  | **≤65 years-old** | **> 65 years-old** |
| --- | --- | --- | --- |
| All-cause mortality | HR (95% CI) | 0.98 [0.82, 1.17] | 1.40 [1.25, 1.57] |
|  | p-value | 0.8111 | 3.77x10^-9^ |
|  | Events/N | (128 / 22,886) | (274 / 19,422) |
| CVD mortality | HR (95% CI) | 0.80 [0.52, 1.25] | 1.73 [1.38, 2.17] |
|  | p-value | 0.3336 | 2.18x10^-6^ |
|  | Events/N | (23 / 22,886) | (53 / 19,422) |
| IHD mortality | HR (95% CI) | 0.72 [0.43, 1.20] | 1.88 [1.39, 2.54] |
|  | p-value | 0.2097 | 4.45x10^-5^ |
|  | Events/N | (17 / 22,886) | (27 / 19,422) |

Results are from Cox proportional hazard regression models with outcomes of interest set as the model outcome (response variable), native T1 is the exposure of interest, and there is adjustment for age, sex, and age x sex. The effect estimates as expressed as HR per 1 SD increase in T1 (i.e., change in hazard of outcome 1SD=35.7ms increase in native T1) with corresponding 95% CI and p-values. CI: confidence interval; HR: hazard ratio; SD: standard deviation; CVD: cardiovascular disease.

**Supplemental Table 5. The associations of native T1 with incident outcomes stratified by median T1**

|  |  | **T1 <median** | **T1 ≥Median** |
| --- | --- | --- | --- |
| Incident CVD (any) | HR [95% CI] | 1.01 [0.89, 1.15] | 1.20 [1.09, 1.33] |
|  | p-value | 0.8683 | 3.15x10^-4^ |
|  | Events/N | (655 / 18,577) | (601 / 18,846) |
| Incident atrial fibrillation | HR [95% CI] | 1.38 [0.97, 1.96] | 1.55 [1.28, 1.88] |
|  | p-value | 0.0695 | 8.55x10^-6^ |
|  | Events/N | (118 / 20,782) | (97 / 20,773) |
| Incident heart failure | HR [95% CI] | 1.17 [0.82, 1.66] | 1.55 [1.32, 1.83] |
|  | p-value | 0.3868 | 1.32x10^-7^ |
|  | Events/N | (103 / 21,027) | (140 / 21,003) |
| Incident stroke | HR [95% CI] | 1.07 [0.77, 1.48] | 1.12 [0.87, 1.44] |
|  | p-value | 0.6976 | 0.3735 |
|  | Events/N | (108 / 20,716) | (107 / 20,740) |
| Incident ischaemic heart disease | HR [95% CI] | 0.98 [0.82, 1.15] | 1.17 [1.01, 1.37] |
|  | p-value | 0.7698 | 0.0382 |
|  | Events/N | (378 / 19,679) | (271 / 20,025) |
| Incident myocardial infarction | HR [95% CI] | 0.94 [0.72, 1.21] | 1.11 [0.84, 1.46] |
|  | p-value | 0.6229 | 0.4706 |
|  | Events/N | (152 / 20,543) | (89 / 20,704) |
| All-cause mortality | HR [95% CI] | 0.97 [0.77, 1.24] | 1.21 [1.03, 1.42] |
|  | p-value | 0.8268 | 0.0236 |
|  | Events/N | (180 / 21,154) | (222 / 21,154) |
| Cardiovascular disease mortality | HR [95% CI] | 1.10 [0.59, 2.04] | 1.19 [0.82, 1.72] |
|  | p-value | 0.7731 | 0.3509 |
|  | Events/N | (30 / 21,154) | (46 / 21,154) |
| Ischaemic heart disease mortality | HR [95% CI] | 0.80 [0.41, 1.54] | 1.32 [0.84, 2.09] |
|  | p-value | 0.4988 | 0.2295 |
|  | Events/N | (19 / 21,154) | (25 / 21,154) |

Results are from Cox proportional hazard regression models with outcomes of interest set as the model outcome (response variable), native T1 is the exposure of interest, and there is adjustment for age, sex, and age x sex. The effect estimates as expressed as HR per 1 SD increase in T1 (i.e., change in hazard of outcome 1SD=35.7ms increase in native T1) with corresponding 95% CI and p-values. CI: confidence interval; HR: hazard ratio; SD: standard deviation.

**Supplemental Table 6. Associations of myocardial native T1 with potential confounders**

| **Outcomes/ confounders** | **Beta** | **95% CI** | **p-value** | **n** |
| --- | --- | --- | --- | --- |
| Heart rate | 0.17* | [0.16, 0.18] | <0.0001 | 37,175 |
| BMI (kg/m^2^) | -0.13* | [-0.14, -0.12] | <0.0001 | 42,307 |
| Hematocrit percentage (baseline) | -0.06* | [-0.07, -0.05] | <0.0001 | 40,314 |

Results are from linear regression models with confounders of interest set as the model outcome and native T1 as the exposure of interest; models are adjusted for age, sex, and age x sex. The effect estimates as expressed as standardised beta coefficients with corresponding standardised 95% CI and p-values. CI: confidence interval

**Supplemental Table 7. Associations of myocardial native T1 with prevalent disease and incident outcomes with additional adjustment for potential measurement confounders**

| **Prevalent outcomes** | **OR [95% CI]** | **p-value** | **Cases / N** |
| --- | --- | --- | --- |
| Any cardiovascular disease | 1.11* [1.07, 1.15] | <0.0001 | (3,939 / 35,361) |
| Any brain disease | 1.12* [1.08, 1.18] | <0.0001 | (2,461 / 35,361) |
| Valvular heart disease | 1.12* [1.02, 1.21] | 0.0108 | (641 / 35,361) |
| Heart failure | 1.47* [1.29, 1.66] | <0.0001 | (217 / 35,361) |
| Non-ischaemic cardiomyopathies | 1.46* [1.18, 1.77] | 0.0003 | (81 / 35,361) |
| Cardiac arrhythmias | 1.19* [1.13, 1.25] | <0.0001 | (1,828 / 35,361) |
| Atrial fibrillation | 1.25* [1.15, 1.36] | <0.0001 | (594 / 35,361) |
| Myocardial infarction | 1.18* [1.09, 1.27] | <0.0001 | (850 / 35,361) |
| Ischaemic heart disease | 1.07* [1.02, 1.12] | 0.0105 | (2,086 / 35,361) |
| Stroke | 1.15* [1.06, 1.24] | 0.0007 | (692 / 35,361) |
| Hypertension | 0.91* [0.89, 0.94] | <0.0001 | (11,542 / 35,361) |
| Diabetes | 1.11* [1.06, 1.17] | <0.0001 | (2,026 / 35,361) |
| High cholesterol | 0.99 [0.96, 1.01] | 0.3098 | (12,320 / 35,361) |
| **Incident outcomes** | **HR [95% CI]** | **p-value** | **Cases / N** |
| Incident CVD (any) | 1.11* [1.05, 1.19] | <0.0001 | (3,939 / 35,361) |
| Incident atrial fibrillation | 1.25* [1.09, 1.45] | 0.0019 | (196 / 34,767) |
| Incident stroke | 1.12 [0.97, 1.30] | 0.1219 | (197 / 34,669) |
| Incident ischaemic heart disease | 1.01 [0.92, 1.10] | 0.8162 | (580 / 33,275) |
| Incident myocardial infarction | 0.97 [0.84, 1.13] | 0.700 | (212 / 34,511) |
| Incident heart failure | 1.45* [1.28, 1.65] | <0.0001 | (208 / 35,144) |
| All-cause mortality | 1.22* [1.09, 1.35] | 0.0003 | (351 / 35,361) |
| Cardiovascular disease mortality | 1.47* [1.17, 1.84] | 0.0009 | (64 / 35,361) |

Results are from logistic regression models for prevalent disease outcomes and Cox proportional hazard regression models for incident event outcomes. The effect estimates as expressed as HR and OR with corresponding 95% CI and p-values. The outcomes of interest are set as the model outcome, native T1 is the exposure of interest, and there is adjustment for age, sex, age*sex, body mass index, hematocrit, and average heart rate. The effect estimates as expressed as HR with corresponding 95% CI and p-values. HR: hazard ratio; CI: confidence interval. *Indicates statistically significant result following multiple testing adjustment with a false discovery rate of 0.05.

STROBE Statement—Checklist of items that should be included in reports of ***cohort studies***

|  | Item No | Recommendation | Section addressed |
| --- | --- | --- | --- |
| **Title and abstract** | 1 | (*a*) Indicate the study’s design with a commonly used term in the title or the abstract | Title |
|  |  | (*b*) Provide in the abstract an informative and balanced summary of what was done and what was found | Abstract |
| Introduction | | |  |
| Background/rationale | 2 | Explain the scientific background and rationale for the investigation being reported | Introduction |
| Objectives | 3 | State specific objectives, including any prespecified hypotheses | Introduction, Methods |
| Methods | | |  |
| Study design | 4 | Present key elements of study design early in the paper | Title, Methods |
| Setting | 5 | Describe the setting, locations, and relevant dates, including periods of recruitment, exposure, follow-up, and data collection | Methods |
| Participants | 6 | (*a*) Give the eligibility criteria, and the sources and methods of selection of participants. Describe methods of follow-up | Methods |
|  |  | (*b*) For matched studies, give matching criteria and number of exposed and unexposed | NA |
| Variables | 7 | Clearly define all outcomes, exposures, predictors, potential confounders, and effect modifiers. Give diagnostic criteria, if applicable | Methods |
| Data sources/ measurement | 8* | For each variable of interest, give sources of data and details of methods of assessment (measurement). Describe comparability of assessment methods if there is more than one group | Methods |
| Bias | 9 | Describe any efforts to address potential sources of bias | Methods |
| Study size | 10 | Explain how the study size was arrived at | Methods, Suppl. Figure 1 |
| Quantitative variables | 11 | Explain how quantitative variables were handled in the analyses. If applicable, describe which groupings were chosen and why | Methods |
| Statistical methods | 12 | (*a*) Describe all statistical methods, including those used to control for confounding | Methods |
|  |  | (*b*) Describe any methods used to examine subgroups and interactions | Methods |
|  |  | (*c*) Explain how missing data were addressed | Methods |
|  |  | (*d*) If applicable, explain how loss to follow-up was addressed | Methods |
|  |  | (*e*) Describe any sensitivity analyses | Methods |
| Results | | |  |
| Participants | 13* | (a) Report numbers of individuals at each stage of study—e.g. numbers potentially eligible, examined for eligibility, confirmed eligible, included in the study, completing follow-up, and analysed | Suppl. Figure 1 |
|  |  | (b) Give reasons for non-participation at each stage | Suppl. Figure 1 |
|  |  | (c) Consider use of a flow diagram | Suppl. Figure 1 |
| Descriptive data | 14* | (a) Give characteristics of study participants (e.g. demographic, clinical, social) and information on exposures and potential confounders | Results, Table 1 |
|  |  | (b) Indicate number of participants with missing data for each variable of interest | Suppl. Figure 1, Table 1 |
|  |  | (c) Summarise follow-up time (e.g., average and total amount) | Results |
| Outcome data | 15* | Report numbers of outcome events or summary measures over time | Table 1 |
| Main results | 16 | (*a*) Give unadjusted estimates and, if applicable, confounder-adjusted estimates and their precision (e.g., 95% confidence interval). Make clear which confounders were adjusted for and why they were included | Table 3, Table 4 |
|  |  | (*b*) Report category boundaries when continuous variables were categorized | Table 2 |
|  |  | (*c*) If relevant, consider translating estimates of relative risk into absolute risk for a meaningful time period | NA |
| Other analyses | 17 | Report other analyses done—e.g. analyses of subgroups and interactions, and sensitivity analyses | Suppl. Table 2-4 |
| Discussion | | |  |
| Key results | 18 | Summarise key results with reference to study objectives | Discussion |
| Limitations | 19 | Discuss limitations of the study, taking into account sources of potential bias or imprecision. Discuss both direction and magnitude of any potential bias | Discussion |
| Interpretation | 20 | Give a cautious overall interpretation of results considering objectives, limitations, multiplicity of analyses, results from similar studies, and other relevant evidence | Discussion |
| Generalisability | 21 | Discuss the generalisability (external validity) of the study results | Discussion |
| Other information | | |  |
| Funding | 22 | Give the source of funding and the role of the funders for the present study and, if applicable, for the original study on which the present article is based | Acknowledgements, Role of funders |

*Give information separately for exposed and unexposed groups.

**Note:** An Explanation and Elaboration article discusses each checklist item and gives methodological background and published examples of transparent reporting. The STROBE checklist is best used in conjunction with this article (freely available on the Web sites of PLoS Medicine at http://www.plosmedicine.org/, Annals of Internal Medicine at http://www.annals.org/, and Epidemiology at http://www.epidem.com/). Information on the STROBE Initiative is available at http://www.strobe-statement.org.
